# Latent Period and Transmission of “*Candidatus* Liberibacter solanacearum” by the Potato Psyllid *Bactericera cockerelli* (Hemiptera: Triozidae)

**DOI:** 10.1371/journal.pone.0093475

**Published:** 2014-03-28

**Authors:** Venkatesan G. Sengoda, W. Rodney Cooper, Kylie D. Swisher, Donald C. Henne, Joseph E. Munyaneza

**Affiliations:** 1 Yakima Agricultural Research Laboratory, Agricultural Research Service, United States Department of Agriculture, Wapato, Washington, United States of America; 2 Department of Entomology, Subtropical Pest Management Laboratory, Texas A&M AgriLife Research, Weslaco, Texas, United States of America; United States Department of Agriculture, United States of America

## Abstract

“*Candidatus* Liberibacter solanacearum” (Lso) is an economically important pathogen of solanaceous crops and the putative causal agent of zebra chip disease of potato (*Solanum tuberosum* L.). This pathogen is transmitted to solanaceous species by the potato psyllid, *Bactericera cockerelli* (Šulc), but many aspects of the acquisition and transmission processes have yet to be elucidated. The present study was conducted to assess the interacting effects of acquisition access period, incubation period, and host plant on Lso titer in psyllids, the movement of Lso from the alimentary canal to the salivary glands of the insect, and the ability of psyllids to transmit Lso to non-infected host plants. Following initial pathogen acquisition, the probability of Lso presence in the alimentary canal remained constant from 0 to 3 weeks, but the probability of Lso being present in the salivary glands increased with increasing incubation period. Lso copy numbers in psyllids peaked two weeks after the initial pathogen acquisition and psyllids were capable of transmitting Lso to non-infected host plants only after a two-week incubation period. Psyllid infectivity was associated with colonization of insect salivary glands by Lso and with Lso copy numbers >10,000 per psyllid. Results of our study indicate that Lso requires a two-week latent period in potato psyllids and suggest that acquisition and transmission of Lso by psyllids follows a pattern consistent with a propagative, circulative, and persistent mode of transmission.

## Introduction

“*Candidatus* Liberibacter solanacearum” (Lso) (Rhizobiales: Rhizobiaceae) is an economically important pathogen of solanaceous crops (Solanales: Solanaceae) in North and Central America and New Zealand [1]–[4]. This bacterium is associated with zebra chip disease of potato (*Solanum tuberosum* L.), which is characterized by striped patterns in tubers that render them unmarketable [4]. Two haplotypes of Lso have been documented in solanaceous crops and designated as A and B [5]–[7]. Both Lso haplotypes are transmitted among solanaceous host plants by the potato psyllid, *Bactericera cockerelli* (Šulc) (Hemiptera: Triozidae) [4], [8]–[9]. Four haplotypes of potato psyllid have been described and appear related to geographic regions in the United States [10]–[13]. The haplotypes have been referred to as Central, Western, Northwestern, and Southwestern [10]–[13]. Mechanisms by which the potato psyllid acquires and transmits Lso are poorly understood.

Results of our previous study indicated that Lso titer in potato psyllids increased for 15 days following the pathogen acquisition and then remained constant through the remaining duration of the study [14]. Lso has been shown to be distributed in all parts of the potato psyllid, including the alimentary canal, salivary glands, and bacteriomes [15]. It is presumed that transmission of Lso to new host plants can only occur after the pathogen has colonized the salivary glands of the vector. Little is known on Lso latent period in the potato psyllid or the relationships among transmission of Lso, Lso titer in the psyllid, and infection of specific psyllid organs. The overall objective of this study was to investigate mode of acquisition and transmission of Lso by the potato psyllid. Specific objectives were to 1) determine the latent period of Lso in potato psyllid, 2) assess the relationship between the latent period of Lso and its copy numbers in psyllids, and 3) assess the relationship between the latent period of Lso and infection of the salivary glands or alimentary canal of the insect.

## Materials and Methods

### Insects and plants

Lso-free and -infected potato psyllid colonies were established at USDA-ARS in Wapato, WA (46° 28’ 10.62” N and 120° 22’ 43.10” W) from insects originally collected from commercial potato fields near Dalhart, TX (36° 00’ 35.38” N and 102° 46’ 40.51” W) in 2007; no collection or import permit was required. Using high resolution melting analysis (Table 1) as described by Swisher et al. [10]–[11] and Chapman et al. [16], the psyllids were determined to be of the Central haplotype (Table 1). The colonies were maintained at 29°C with a 16:8 (L:D) h photoperiod and 50% relative humidity (RH) in a controlled environment room. Samples of insects from both colonies were regularly tested for the presence or absence of Lso using conventional polymerase chain reaction (cPCR).

**Table 1:** Primers and probes used in this study.

Primers/probes	Sequence 5′ - 3′	Location	Size (bp)	References
**OA2** a	GCGCTTATTTTTAATAGGAGCGGCA	16S rDNA	1168	Liefting et al.[2]
**OI2c** a	GCCTCGCGACTTCGCAACCCAT	16S-23S rDNA	-	Jagoueix et al. [20]
**CL-ZC-F** b	TCGGATTTAGGAGTGGGTAAGTGG	Outer membrane protein	185	Crosslin et al. [21]
**CL-ZC-R** b	ACCCTGAACCTCAATTTTACTGAC	Outer membrane protein	-	Crosslin et al. [21]
**CL-ZC-P** b	6Fam-TTGGCACCATGAACCGCAGAAACACTAAT-Tamra		-	Crosslin et al. [21]
**28SF** c	TCGGTCGTTTCCGTTGGT	28S rDNA	67	Sengoda et al. [14]
**28SR** c	GGCGCACACGAATCAACAT	28S rDNA	-	Sengoda et al. [14]
**28SP** c	6Fam-ACGCGACCAGCGTTGCGTCTTC-Tamra		-	Sengoda et al. [14]
**Lso-SSR-1F** d	TTATTTTGAGATGGTTTGTTAAATG	Phosphatidylserine synthase	180/240	Lin et al. [6]; Wen et al. [7]
**Lso-SSR-1R** d	TATTATCATTCTATTGCCTATTTCG	Phosphatidylserine synthase		Lin et al. [6]; Wen et al. [7]
**Lso FISH probe** e	Alexa488- GCCTCGCGACTTCGCAACCCAT	16S-23S		Jagoueix et al. [20]; Cooper et al. [15]
**CO1 F3** f	TACGCCATACTAGCAATCGG	Cytochrome oxidase	94	Swisher et al. [10]
**BB bc melt CO1 reverse** f	TGAAATAGGCACGAGAATCAA	Cytochrome oxidase		Chapman et al. [16]

a Conventional PCR of Lso.

b Quantitative real-time PCR of Lso.

c Quantitative real-time PCR of potato psyllid.

d Lso haplotype differentiation, 240 bp  =  Lso haplotype A; 180 bp  =  Lso haplotype B.

e Fluorescence *in situ* hybridization of Lso.

f Psyllid haplotyping primers.

Potato, tomato (*Solanum lycopersicum* L.), and sweet potato (*Ipomoea batatas* (L.) Lam) were used in the present study as host plants for the potato psyllid. Plants were grown in a greenhouse in 0.5-L pots (Kord Products, Toronto, Ontario, Canada) filled with a soil media consisting of 86% sand, 13.4% peat moss, 0.5% Apex time release fertilizer (J. R. Simplot Co., Lathrop, CA), and 0.1% Micromax micronutrients (Scotts Co., Marysville, OH). Sweet potato ‘White Delight’ plants were established from the propagation of stem cuttings, ‘Atlantic’ potato plants were grown from certified disease-free tubers, and ‘Early Girl’ tomato plants were grown from seed (Ed Hume, Inc., Puyallup, WA). Lso-inoculum plants were generated by confining ten Lso-infected adult psyllids to one-month old potato or tomato plants for three days. The insects were removed from plants by fumigation with methyl bromide, and then the plants were maintained in a greenhouse until foliar symptoms associated with Lso infection were observed [14]. Following inoculation, foliar symptoms were observed on potato and tomato inoculum plants after about one and two months, respectively, and Lso infection was confirmed by both cPCR and quantitative real-time PCR (qPCR) prior to conducting experiments. Our previous study [14] showed that Lso titer in tomato plants was 200- to 400-fold higher than in potato plants; however, there was no difference in Lso titer in potato psyllid adults two weeks following acquisition of the bacterium from either infected potato or tomato plants, regardless of acquisition access period. Therefore, we did not quantify Lso titer in inoculum plants prior to conducting the acquisition and inoculation experiments in the present study but rather relied on visual Lso infection symptoms in the inoculum plants and used cPCR to confirm infection. Although a suitable host for the potato psyllid, sweet potato is not a host to Lso [14]; thus, this plant species was used to maintain psyllids following acquisition of the bacterium from the inoculum plants.

### Latent period study

The experimental design to determine time between Lso acquisition and effective transmission by the potato psyllid (latent period) was similar to that described by Sengoda et al. [14]. Non-infected psyllids were exposed to Lso by releasing the insects onto plants in cages (#1462W BugDorm-2, BioQuip Products, Rancho Dominguez, CA) kept in a greenhouse maintained at 24-28°C with supplemental lighting to provide a 16:8 (L:D) h photoperiod. Each cage contained either five potato or tomato inoculum plants. After the acquisition access periods (AAP) of 24 or 72 h, insects were removed from the inoculum plants using an aspirator and transferred to sweet potato plants. Since sweet potato is not a host for Lso, insects could acquire Lso only from the 24- or 72-h exposure to the inoculum (potato or tomato) plants [14]. Beginning immediately after removing the insects from the inoculum plants, samples of 30 insects were collected from sweet potato each week for 3 weeks (Lso incubation period). At each collection, (0, 1, 2, and 3 weeks of incubation period), each insect was individually confined to a three-week old non-infected potato plant for 24 h. Positive controls were established by confining single insects obtained from an Lso-infected colony to each of 5 plants for 24 h. After the 24-h inoculation access period, the insects were collected and were either stored at –20°C pending PCR analysis or were immediately processed by fluorescence *in situ* hybridization to track Lso movement. The inoculated potato plants were maintained in the greenhouse and periodically observed for foliar symptoms of Lso infection. Beginning one month after inoculation and every two weeks thereafter, samples of the leaf, petiole, and stem tissues were collected from each plant to test for the presence of Lso using both cPCR and qPCR until the plants were dead or the bacterium was detected. It was necessary to test plants for Lso every two weeks because infected plants often decline and die quickly before they can produce tubers. Following death of the above-ground portions of the plants, any produced tubers were assessed for the presence or absence of zebra chip symptoms as described by Munyaneza et al. [8]–[9].

The two Lso-tracking analyses – qPCR analysis and fluorescence *in situ* hybridization – were each conducted twice (two trials) with different cohorts of insects and different inoculum plants. Each trial included 8 to 10 insects per combination of AAP (24 or 72 h), inoculum host (potato or tomato), and week of incubation period (0, 1, 2, or 3 weeks). The fluorescence *in situ* hybridization trials included two non-infected psyllids for each week of the incubation period, to serve as negative controls.

### Nucleic acid extractions and polymerase chain reaction (cPCR and qPCR)

Total DNA was extracted from plants and insects using a cetyltrimethlyammonium bromide (CTAB) buffer extraction method [17]–[19]. About 400 mg of leaf and plant tissues were macerated in BioReba sample bags with 1 ml of extraction buffer (100 mM Tris-HCl, pH 8.0, 50 mM EDTA, 500 mM NaCl, and 10 mM mercaptoethanol) using a Homex 6 homogenizer (BioReba, Reinach, Switzerland). Following this, 300 μl of macerate was collected, mixed with 80 μl of lysozyme (50 mg/ml in 10 mMTris-HCl, pH 8.0, Sigma-Aldrich, St. Louis, MO), and incubated for 30 min at 37°C. After incubation, 500 μl of CTAB buffer (2% CTAB, 1.4MNaCl, 20 mM EDTA, 100 mM Tris-HCl, pH 8.0, and 0.2% mercaptoethanol) was added to each sample of macerated plant tissue. Whole insects suspended in 600 μl of CTAB buffer were macerated using a micropestle. Plant and insect macerates in CTAB buffer were incubated for 30 min at 65°C, then maintained at room temperature for 3 min before adding 500 μl (plant samples) or 600 μl (insect samples) of ice-cold chloroform. After vortexing the samples, the samples were centrifuged at 13,000 × *g* for 10 min and the resulting aqueous layer was added to 500 μl of isopropanol and glycogen (1 μl/ml). DNA was recovered by centrifugation at 16,000 × *g* for 10 min after maintaining the tubes on ice for 20 min. The pellets were washed with ice-cold 70% ethanol, centrifuged at 13,000 × *g* for 3 min, and allowed to air dry. Plant DNA was resuspended in 100 μl of sterile water whereas insect DNA was resuspended in 50 μl of sterile water.

Genomic DNA from insects was quantified using Quant-iT™ PicoGreen ® dsDNA Reagent and Kits (Molecular probes, Cat. No: P11496). The fluorescence (excitation ∼480 nm, emission ∼520 nm) was measured using Thermo Scientific Fluoroskan Ascent Microplate Fluorometer. Final DNA concentrations were adjusted to 2 ng/μl and 5 μl were used for both cPCR and qPCR (10 ng/reaction of insect DNA).

Initially, plants and insect samples were tested for Lso using cPCR primers OA2/OI2c targeting the 16S rDNA region (Table 1) [1]–[2], [20]. Amplifications were performed in 50 μl reactions with Green Go *Taq* Polymerase (Promega, Madison, WI) according to the manufacturer’s instructions. For each reaction, 20 pmol of each primer and 2 μl of DNA extract were added and incubated under the following conditions: initial denaturation for 3 min at 94°C and then amplification for 30 sec at 94°C, 30 sec at 65°C, 1 min at 72°C for 39 cycles, followed by a final 5 min incubation at 72°C (MJ Research). PCR products were separated on 1.5% agarose gels containing ethidium bromide for visualization. To determine which types of Lso the psyllids had acquired, Lso haplotyping was performed using Lso-SSR-1F/Lso-SSR-1R according to Lin et al. [6] and Wen et al. [7] (Table 1), and cPCR products were separated on 2.0% agarose gels containing ethidium bromide. To assess Lso acquisition rate and copy numbers, qPCR was performed using primers and probes targeting the Lso outer membrane protein and the psyllid 28S rDNA(Table 1) [14], [21] with 3 replications per sample. Standard curve construction and gene quantification for the Lso outer membrane protein and the psyllid 28S rDNA (Table 1) were performed as described by Sengoda et al. [14] and Marzachi and Bosco [22]. The qPCR with Lso or 28S rDNA primers and probes consisted 12.5 μl TaqMan® Universal PCR Master Mix (Applied Biosystems; Roche Diagnostics, Indianapolis, IN), 2.5 μl of each primer (9 μM), 2.5 μl of labeled probe (2.5 μM), and 5 μl nucleic acid extracts (10 ng of psyllids genomic DNA). Reactions were amplified on Chromo4 (BioRad) with the following cycling conditions: 50°C for 2 min, 95°C for 10 min, then 40 cycles of 95°C for 15 sec and 60°C for 60 sec. Lso copy numbers were calculated using Opticon 3 software with a Ct cut off value of 37 and expressed per 10 ng of psyllid genomic DNA. Interplate reproducibility was assessed by running qPCR of a known quantity of genomic DNA from Lso-infected psyllids (4.5 and 0.45 ng targeting the Lso outer membrane protein) or genomic DNA from Lso-free psyllids (4.5, 0.45, and 0.045 ng targeting the psyllid 28S rDNA), along with experimental samples and appropriate positive and negative controls. The coefficient of variation of the average Ct values of different plates was compared for both Lso and psyllid 28S rDNA.

The CL-ZC-F/CL-ZC-R (185 bp) and 28SF/R (67 bp) amplicons obtained by cPCR were cloned using the TOPO TA cloning kit (Invitrogen, Carlsbad, CA) with TOP 10 *Escherichia coli* chemically competent cells. Plasmid DNA was extracted from selected colonies using the QIAprep spin mini prep kit (QIAGEN, Valencia, CA), and the DNA clones were sequenced by MC Laboratories (MCLab, San Francisco, CA). The Lso outer membrane protein plasmid, Lso-OMP, and potato psyllid 28S rDNA and 28SF/28SR fragments were confirmed by BLASTn analysis. The Lso-OMP plasmid and Lso-free psyllid genomic DNA were used to construct standard curves as described below.

The Lso standard curve was constructed using Lso-OMP plasmids for quantification of Lso copies in post-acquisition psyllids and inoculum plants. The DNA copy numbers were calculated as follows, assuming the average weight of a nucleotide base pair was 660 Daltons:

DNA (copies/μl)  =  DNA (ng/μl)/(DNA (bp) * 1×10^9^ (ng/g) * 660 (Da/bp)/6.022×10^23^ (copies/mol). Lso-OMP plasmids were diluted to final concentrations of 2240000 copies/μl, 224000 copies/μl, 22400 copies/μl, 2240 copies/μl, 224 copies/μl, 22.4 copies/μl, 2.24 copies/μl in 2ng/μl or 40ng/ul of Lso-free psyllid genomic DNA. In each dilution, 5μl was loaded to get 10 fold serial dilution final concentrations of 11200000, 1120000, 112000, 11200, 1120, 112, and 11.2 Lso copies. Lso-free psyllid genomic DNA was used to construct the insect standard curve. Insect genomic DNA was diluted in water to a final concentration of 5 ng/μl, 0.5 ng/μl, 0.05 ng/μl, 0.005 ng/μl, and 0.0005 ng/μl. For each dilution, 5 μl was loaded to get final concentrations of 25 ng, 2.5 ng, 0.25 ng, 0.025 ng, and 0.0025 ng. Identical fluorescence threshold and baseline settings were used for comparability of results. Amplification efficiency was calculated using the following formula: E  =  10∧-(1/slope).

### Fluorescence *in situ* hybridization

Fluorescence *in situ* hybridization was performed using the methods described in Cooper et al. [15] and Ammar et al. [23]. An adult psyllid anesthetized with CO_2_ was mounted on a glass microscope slide with its ventral side facing up using double-sided tape. A drop of phosphate buffered saline (Fisher Scientific, Pittsburgh, PA) was placed over the insect and held in place by cohesion. Using two #5 forceps (D’Outils Dumont SA, Montignez, Switzerland), the salivary glands were removed after gently pulling the psyllid’s head away from the body. Both pairs of primary and accessory salivary glands were then transferred to a circle drawn with an Aquahold Barrier PAP pen (Scientific Devise Laboratory, Des Plaines, IL) on a Tissue Tack Microscope slide (Polysciences Inc., Warrington, PA). After removing the salivary glands, the ventral plate of the insect was removed and the alimentary canal was transferred to a separate Aquahold Barrier circle.

**Figure 1:**
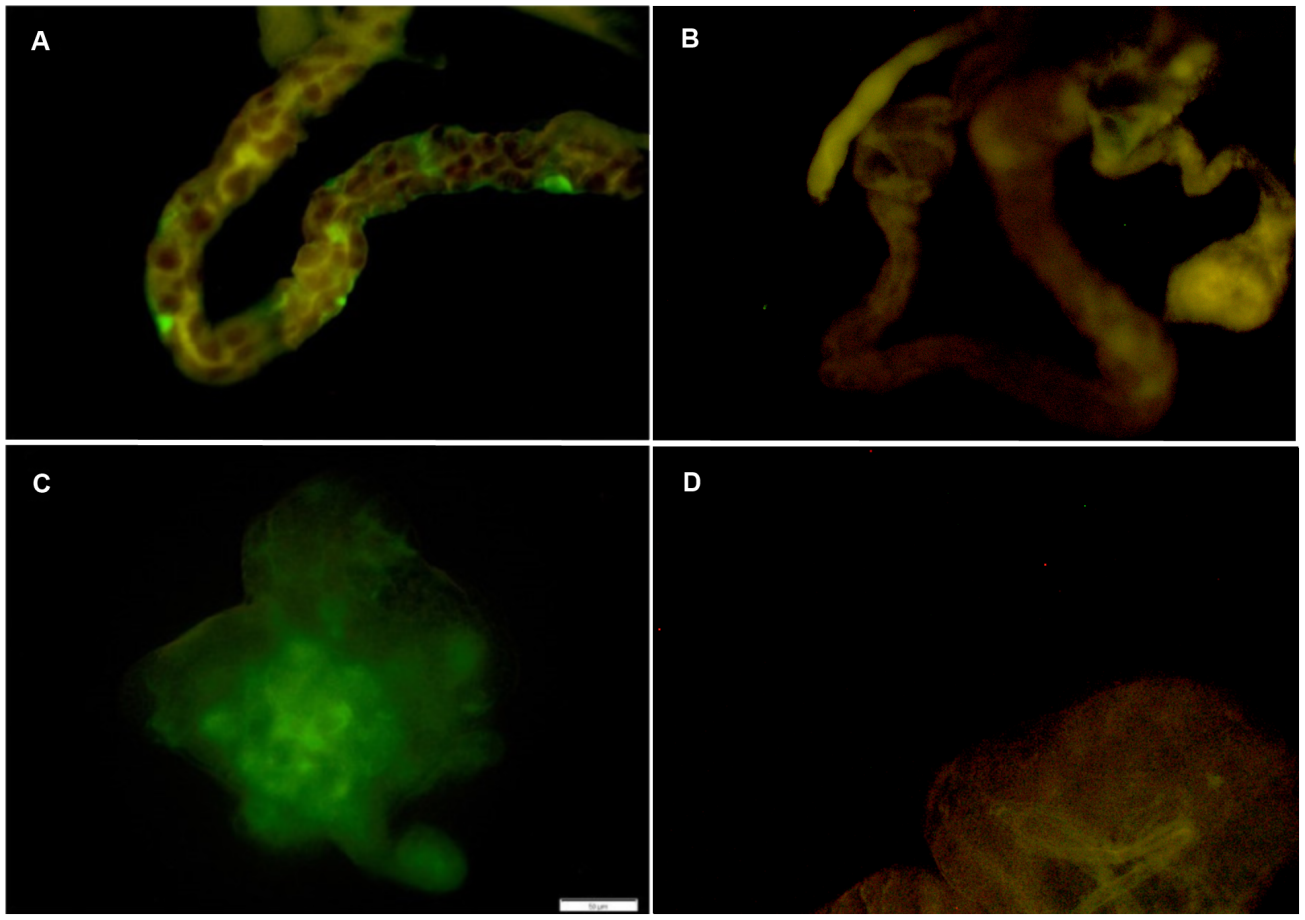
Lso infection in the (A) alimentary canal and (C) the salivary glands of potato psyllids. Green fluoresence indicates the presence of Lso while non-infected tissues appear yellow. Lso non-infected psyllids showed no green fluoresence in the (B) alimentary canal and (D) the salivary glands.

The dissected tissues were air-dried at room temperature. The slides were then maintained for 3-5 min on a slide warmer set at 50°C to adhere the tissues to the slides. Samples were fixed in Carnoy’s solution (Electron Microscopy Sciences, Hatfield, PA) for 1 h, briefly rinsed in 100% ethanol, and washed three times for 20 min in hybridization buffer consisting of 20 mM Tris-HCl (pH 8.0) (Fisher Scientific), 0.9 M NaCl (Fisher Scientific), 0.01% sodium dodecyl sulphate (Indofine Chemical Company, Hillsborough, NJ), and 30% formamide (Fisher, Scientific). Samples were hybridized overnight with 250 pmoles/ml of HPLC-purified oligonucleotide probe labeled with Alexa Fluor 488 on the 5-prime end (Table 1) (Invitrogen, Carlsbad, CA) and dispersed in hybridization buffer. During probe hybridization, samples were kept under humid conditions within an environmental chamber (Percival Scientific, Inc., Perry, IA) maintained at 25 ± 0.5°C with the lights off. After hybridization, samples were briefly washed in hybridization buffer, followed by two washes for 20 min in hybridization buffer, and one 20-min wash in tris-buffered saline (Fisher Scientific). The presence of Lso was detected at 200 or 400× using a fluorescence microscope (Zeis Axioskop 40 FL, Carl Zeiss USA, Thornwood, NY) with Zeiss filter-set 09 (excitation wavelength = 450-490 nm, beam splitter = 510 nm, and emission wavelength = 515 nm). Infected tissues fluoresced in green whereas non-infected tissues appeared yellow (Fig. 1). Occasionally, low-level white light was used to position the slides or to verify the absence of cuticle fragments, which auto-fluoresced green and appeared similar to the fluorescence of the Alexa Fluor 488 probe. Samples were photographed using a DP25 camera mounted to the microscope and operated using the CellSens software (Olympus America Inc., Central Valley, PA).

### Statistical analysis

Lso copy numbers in psyllids were compared among combinations of AAP, incubation period, and inoculum host using the GLIMMIX (Restricted Maximum Likelihood) procedure of SAS 9.3 (SAS Institute 2012). AAP, Lso incubation period, inoculum host, and the main effect interactions were included as the fixed effects. Trial was included as the random variable. Corrected denominator degrees of freedom were obtained using the Kenward-Roger adjustment (DDFM = KR option of the MODEL statement). Residual and normal quantile-quantile plots were used to examine data for evidence of heterogeneity of variance and non-normality of errors, respectively. Based on these plots, the DIST = LOGN option was included in the MODEL statement. Where differences among fixed effects were indicated, differences among means were compared using the ADJUST = SIMULATE option of the LSMEANS statement.

Acquisition and transmission of Lso by potato psyllids were assessed in separate analyses using logistic regression (PROC GLIMMIX). The dependent variable for analysis of Lso acquisition was the number of infected psyllids divided by the total number of psyllids whereas the dependent variable for analysis of Lso transmission was the number of infected plants divided by the total number of plants in each trial. Logistic regression (PROC GLIMMIX) was also used to assess localized infection of psyllid salivary glands and alimentary canals observed using fluorescence *in situ* hybridization. The dependent variables were the number of psyllids with infected salivary glands or infected alimentary canals divided by the total number of psyllids tested in each trial. In all four analyses, the fixed effects were AAP, incubation period, inoculum host, and each main effect interaction. Wald 95% confidence intervals for odds ratios were used to compare means when significant main effect interactions were observed.

To assess the relationship between Lso copy numbers in psyllids and plant infection, Lso copy numbers were categorized into six groups based on Lso copy distribution: 1) 0 copies, 2) 1 to 1000 copies, 3) >1000 to 10,000 copies, 4) >10,000 to 100,000 copies, 5) >100,000 to 1 million copies, and 6) >1 million copies. The probability of plants becoming infected by psyllids with Lso copies in each respective population size class was compared using logistic regression as described above with the proportion of infected plants divided by the total number of plants as the dependent variable, and Lso population size-class as the fixed effect.

Separate contingency table analyses (PROC FREQ of SAS 9.3) were used to determine whether colonization of the alimentary canal or salivary glands by Lso was associated with transmission of Lso by the insect. Contingency table analysis was also used to compare Lso latent period in insects and the proportion of plants infected with Lso haplotype A versus haplotype B. Trial was controlled in each contingency table analysis, and statistical differences were assessed based on the Conchran-Mantel-Haenszel row mean score statistic [24].

## Results

### Zebra chip disease evaluation in potato plants and tubers

Foliar and tuber symptoms associated with Lso infection consistent with those described by Munyaneza [4], Munyaneza et al. [8]–[9], and Sengoda et al. [25] were observed in potato plants exposed to psyllids that effectively acquired Lso and in which the bacterium completed the latent period. About 29.6% of the plants showed foliar and tuber symptoms of zebra chip 3 to 4 weeks after inoculation and were characterized by plant stunting, shortened internodes, yellowing and purpling of leaves, and upward curling of leaves [4]. Also, tubers produced by Lso-infected plants exhibited typical symptoms of zebra chip disease [4]. Between 60 and 80% of the positive control plants (plants infested with psyllids from the Lso-infected laboratory colony) developed foliar symptoms associated with Lso infection. The different assessment methods of Lso infection (foliar symptoms, tuber symptoms, cPCR and qPCR diagnosis) were highly consistent; 100% of plants that exhibited foliar and tuber symptoms associated with Lso infection and zebra chip disease also tested positive for the bacterium by PCR.

### Lso copy numbers

The qPCR targeting the outer membrane protein of Lso generated linear regression lines with a slope between –3.31 and 3.37 (3.34±0.022) with amplification efficiencies of 98.0–99.5%. The qPCR targeting the psyllid 28S rDNA, had linear regression lines with a slope between –3.29 and 3.33 (3.31±0.016) with an amplification efficiency of 100%. The coefficient of variation of the average Ct values was 0.01 (1%) for both Lso (4.5 and 0.45 ng of Lso-infected DNA) and psyllid 28S rDNA (4.5, 0.45, and 0.045 ng of Lso-free psyllid genomic DNA), suggesting that qPCR is highly reproducible and consistent with the previous report by Sengoda et al. [14].

**Figure 2:**
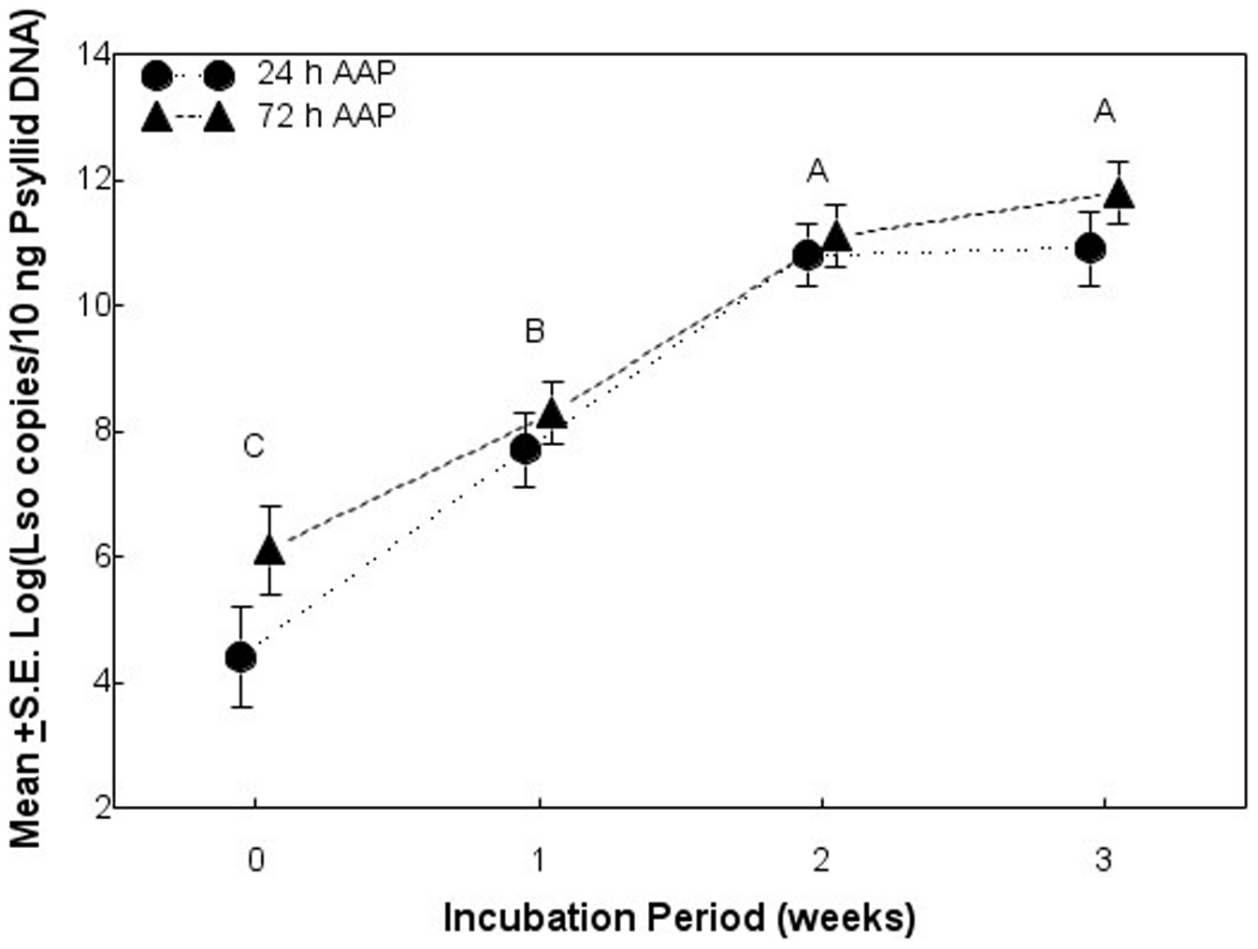
Relationship between acquisition access period, incubation period, and Lso copy numbers in potato psyllids. Different letters indicate significant differences in Lso copy numbers among weeks regardless of acquisition access period.

Lso copy numbers varied in insects subjected to different AAPs (24 versus 72 h) and held for different incubation periods (0, 1, 2, and 3 weeks post-inoculation) (Table 2). Inoculum host (potato versus tomato) did not influence Lso copy numbers in psyllids. The lack of significant main effect interactions indicated that the effects of AAP on Lso copy numbers were independent of weeks of incubation period and inoculum host, and the effects of incubation period were independent of AAP and inoculum host (Table 2). Lso copy numbers were generally higher in insects subjected to a 72-h AAP compared with those subjected to a 24-h AAP. The mean log Lso copy numbers (±S.E.) for psyllids subjected to 24 and 72-h AAP was 8.5 (±0.31) and 9.3 (±0.27), respectively, regardless of week of incubation period. In addition, Lso copy numbers increased from week 0 to week 2 of the incubation period, but did not differ between weeks 2 and 3 (Fig. 2).

**Table 2:** Statistical analyses examining Lso copy numbers in psyllids, the proportion of psyllids and plants infected with Lso, and the proportion of psyllids with infected alimentary canals and salivary glands.

Model Effect	Lso copy numbers in Psyllids^a^	Proportion of psyllids infected with Lso^a^	Proportion of plants infected with Lso^a^	Proportion of psyllids with infected alimentary canals^b^	Proportion of psyllids with infected salivary glands^b^
**Acquisition Access Period (AAP)**	*F_1, 160_ = *4.2; *P* = 0.04	*F_1, 15_* = 9.4; *P<*0.01	*F_1, 15_*<0.14; *P = *0.25	*F_1, 16_* = 0.2; *P = *0.71	*F_1, 16_* = 0.1; *P = *0.99
**Incubation period**	*F_3, 160_ = *40.3; *P*<0.01	*F_3, 15_* = 5.0; *P = *0.01	*F_3, 15_* = 16.8; *P<*0.01	*F_3, 16_* = 0.7; *P = *0.57	*F_3, 16_* = 5.2; *P = *0.01
**AAP× Incubation period**	*F_3, 160_ = *0.5; *P* = 0.70	*F_3, 15_* = 1.3; *P = *0.32	*F_3, 15_<*0.5; *P = *0.68	*F_3, 16_* = 0.7; *P = *0.57	*F_3, 16_* = 0.2; *P = *0.93
**Host Plant**	*F_1, 160_ = *0.1; *P* = 0.75	*F_1, 15_* = 2.2; *P = *0.16	*F_1, 15_<*0.1; *P = *0.87	*F_1, 16_* = 1.6; *P = *0.23	*F_1, 16_* = 0.1; *P = *0.99
**AAP × Host**	*F_1, 160_ = *0.2; *P* = 0.65	*F_1, 15_* = 0.1; *P = *0.98	*F_1, 15_*<0.2; *P = *0.67	*F_1, 16_* = 0.1; *P = *0.99	*F_1, 16_* = 0.1; *P = *0.99
**Incubation period × Host**	*F_3, 160_ = *1.6; *P* = 0.20	*F_3, 15_* = 1.5; *P = *0.26	*F_3, 15_* = 0.2; *P = *0.92	*F_3, 16_* = 1.6; *P = *0.24	*F_3, 16_* = 0.1; *P = *0.96
**APP× incubation period × Host**	*F_3, 160_ = *0.8; *P* = 0.51	*F_3, 15_* = 0.4; *P = *0.76	*F_3, 15_* = 0.1; *P = *0.98	*F_3, 16_* = 0.8; *P = *0.52	*F_3, 16_* = 0.6; *P = *0.62

a Presence of Lso assessed using quantitative real-time PCR.

b Presence of Lso assessed using fluorescence *in situ* hybridization.

### Acquisition and transmission of Lso

**Figure 3:**
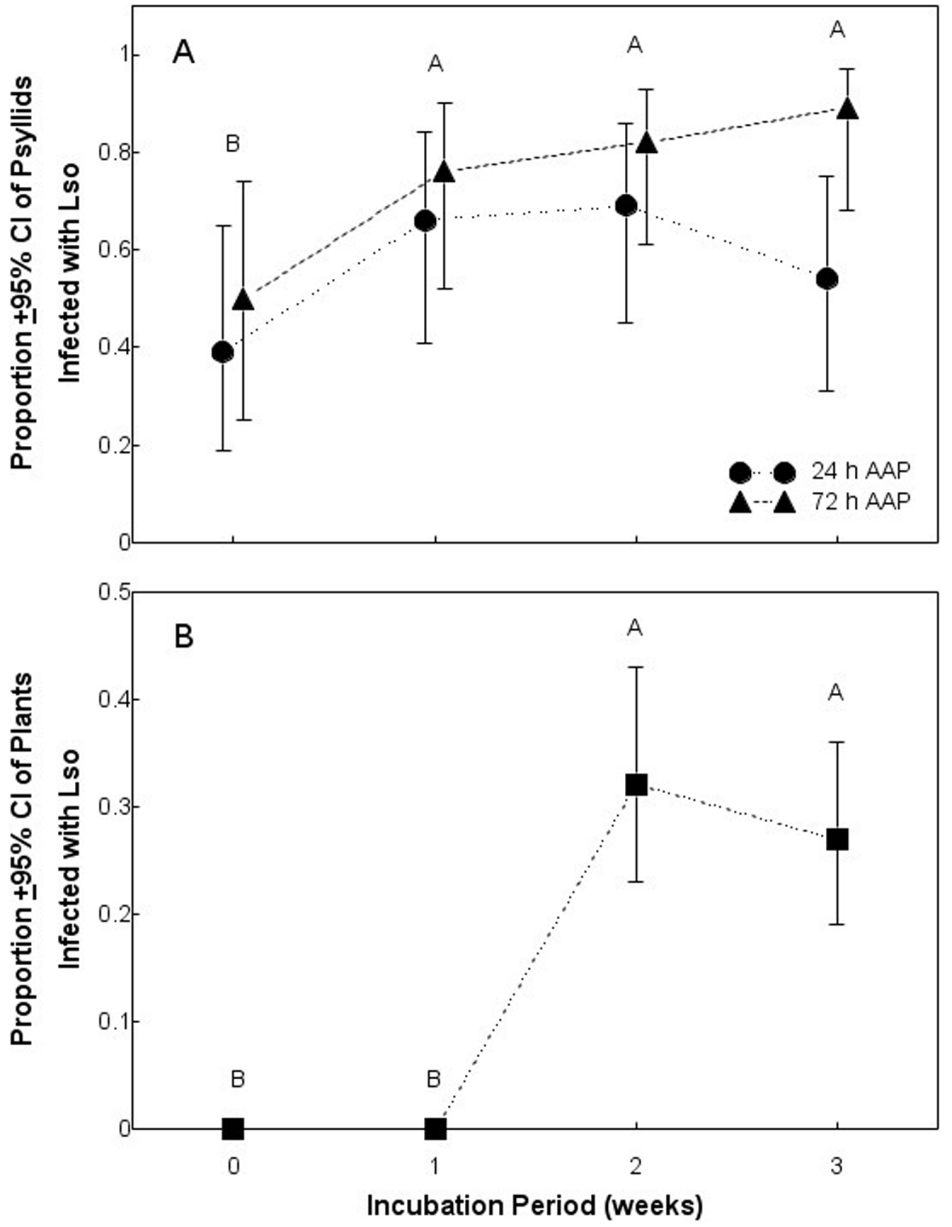
Proportion of potato psyllids (A) or plants (B) to test positive for Lso using quantitative real-time PCR. Different letters indicate significant differences among weeks, regardless of acquisition access period.

The proportion of psyllids that tested positive for Lso by qPCR differed between those subjected to 24 and 72-h AAP, and among psyllids held for different incubation periods (Table 2). Inoculum plant host did not influence the proportion of psyllids that tested positive for Lso, and there were again no main effect interactions (Table 2). The mean (±95% confidence intervals) proportion of psyllids exposed to Lso-infected plants for a 24- and 72-h AAP and tested positive for Lso by qPCR was 0.57 (0.392 – 0.734) and 0.77 (0.602 – 0.876), respectively, regardless of week of incubation period. Significantly fewer psyllids tested positive for Lso on week 0 of the incubation period than on weeks 1 through 3 (Fig. 3A).

Lso transmission rates differed significantly among weeks of incubation period, but did not differ between insects subjected to different AAPs or inoculum plant hosts (Table 2). The lack of significant main effect interactions indicated that the effects of incubation period on Lso transmission were independent of AAP and inoculum host (Table 2). None of the plants exposed to insects during weeks 0 or 1 tested positive for Lso using both cPCR and qPCR, but nearly 30% of plants exposed to insects during weeks 2 and 3 were infected (Fig. 3B), suggesting that the latent period for Lso in an adult potato psyllid is about 2 weeks. It is important to note that contingency table analysis did not indicate significant differences in the proportion of insects or plants infected with Lso haplotype A versus Lso haplotype B between 2 or 3 weeks of incubation period (row mean score  =  0.06; d.f. = 1; *P* = 0.79).

### Lso infection of psyllid alimentary canal and salivary glands

**Figure 4:**
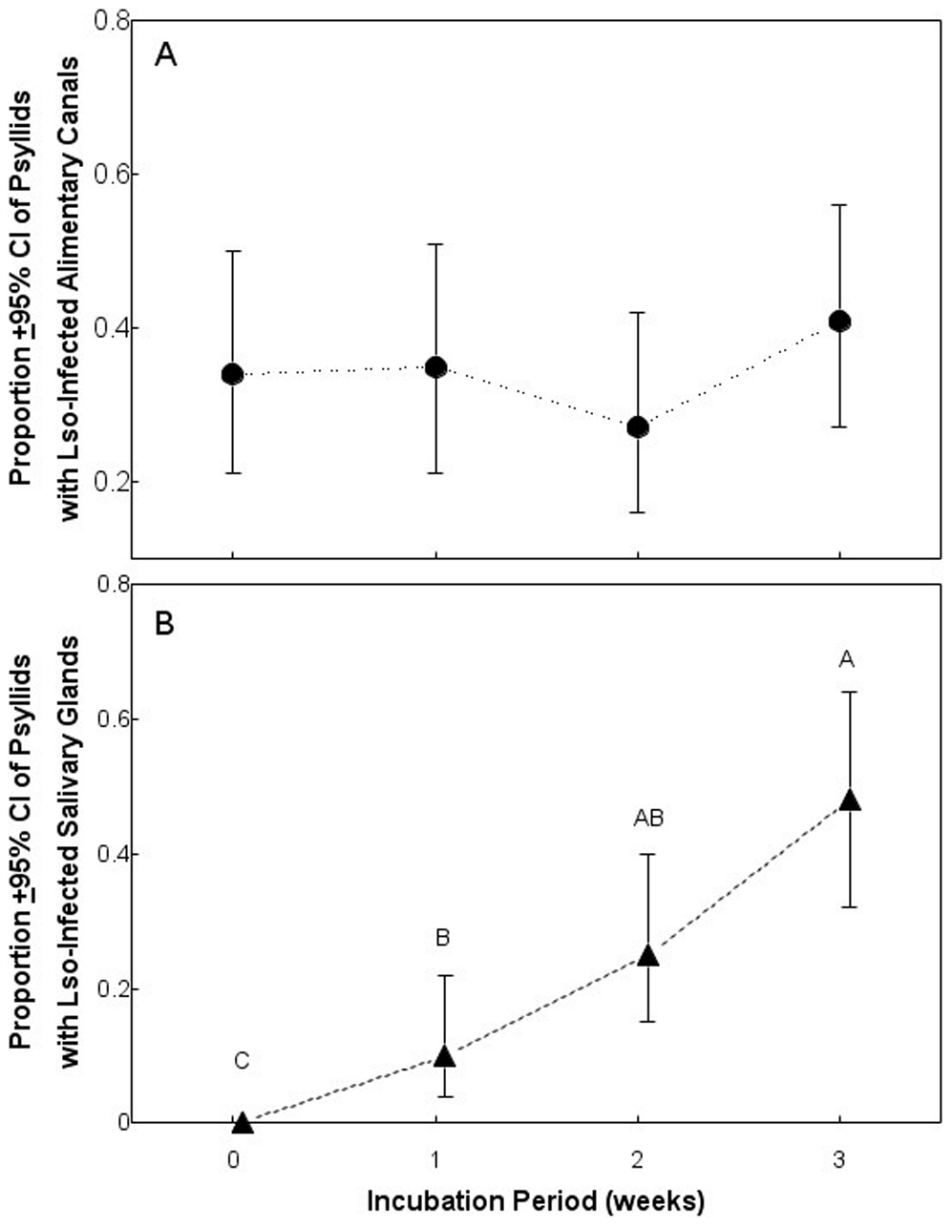
Proportion of psyllids with an infected alimentary canal (A) or salivary glands (B). Different letters indicate significant differences in infection rates among weeks.

There were no statistical effects of AAP, incubation period, or host plant on the probability of detecting Lso infection using fluorescence *in situ* hybridization in the psyllid alimentary canal, and there were no significant interactions among factors (Table 2; Fig. 4A). AAP and inoculum host did not influence the probability of detecting Lso infection in psyllid salivary glands, but the proportion of salivary glands infected with Lso did increase with increasing length of the incubation period (Table 2; Fig. 4B). The lack of significant interactions among factors indicated that the effects of incubation period on colonization of salivary glands by Lso were independent of AAP, inoculum host, and the AAP by inoculum host interaction.

### Relationships between Lso infection of psyllids and transmission of Lso to host plants

**Figure 5:**
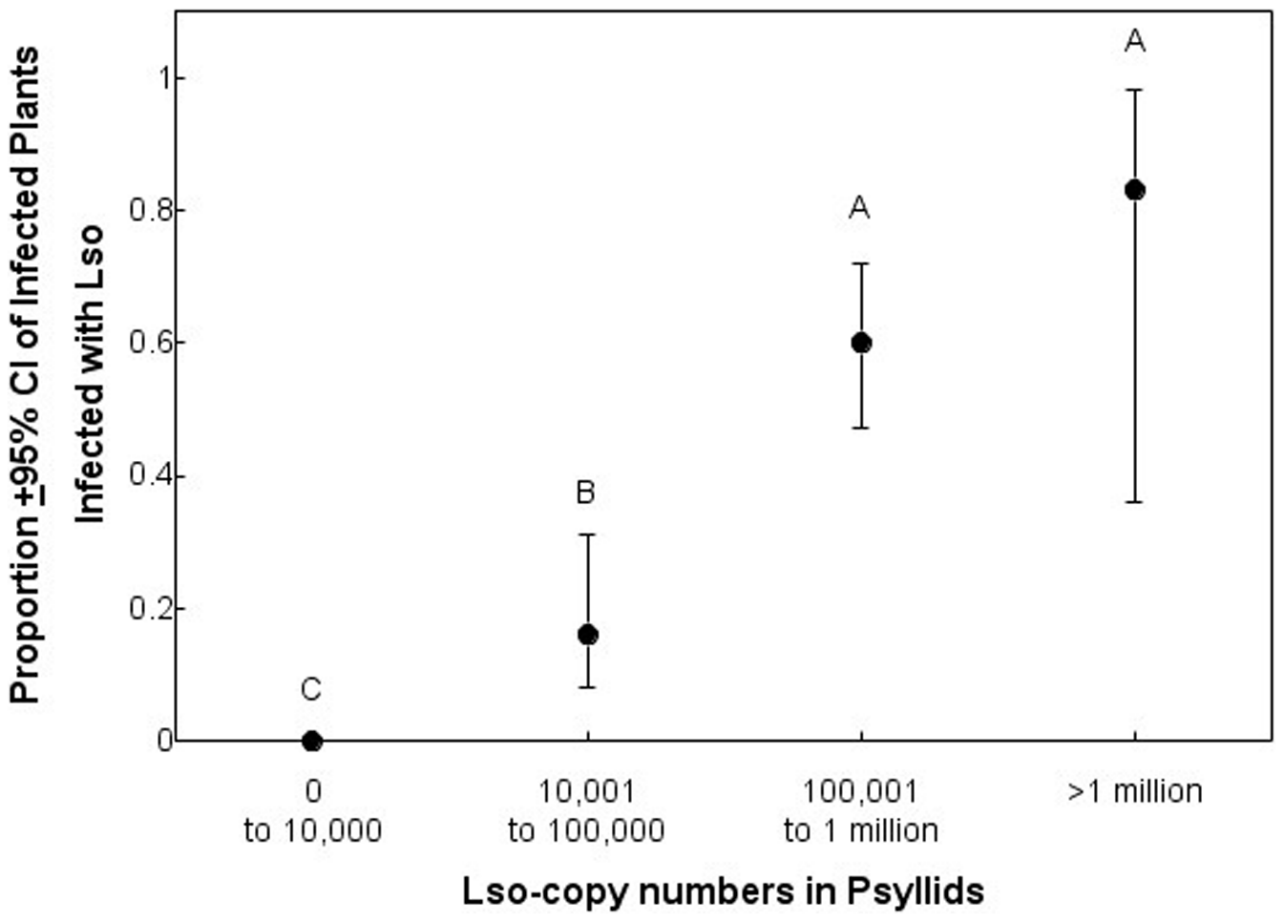
Relationship between Lso copy numbers in psyllids and transmission rates to host plants. Different letters indicate significant differences among Lso copies groups.

To determine the Lso copy numbers required for successful transmission of Lso to the host plant, Lso copies were empirically categorized into 6 groups as described in the materials and methods. Logistic regression showed that Lso transmission was dependent upon Lso copy numbers (*F* = 4.0; d.f. = 5, 106; *P*<0.01). No psyllids with fewer than 10,000 Lso copies transmitted Lso to their host plants, and the probability of plants becoming infected increased with increasing Lso copies in psyllids with more than 10,000 copies (Fig. 5).

On average, fluorescence *in situ* hybridization analysis showed that 17.8% of psyllids with infected alimentary canals and 9.5% of psyllids with non-infected alimentary canals transmitted Lso to their host plants. Contingency table analysis did not indicate that transmission of Lso differed between psyllids with infected and non-infected alimentary canals (row mean score  =  1.8; d.f. = 1; *P* = 0.18). Transmission of Lso differed between psyllids with infected and non-infected salivary glands (row mean score  =  4.5; d.f. = 1; *P* = 0.03) with 25% of psyllids with infected salivary glands successfully transmitting Lso to host plants compared with 9.3% of psyllids with non-infected salivary glands. Categories with zeros excluded from the statistical analysis.

Contingency table analysis did not indicate significant differences in the proportion of plants infected with Lso-haplotype A versus haplotype B between weeks of latent period (row mean score  =  0.06; d.f. = 1; *P* = 0.79). Lso-haplotype B infected 82.9% of plants whereas haplotype A infected only 17.1% of plants, regardless of week of latent period. All inoculum plants tested positive for both Lso haplotype A and Lso haplotype B (data not shown).

## Discussion

Results of the present studies were consistent with those from a number of previous studies. As also reported by Sengoda et al. [14], Lso copy numbers were greater in psyllids subjected to a 72-h AAP compared with those allowed a 24-h AAP. Also consistent with results reported by Sengoda et al. [14], Lso copy numbers in psyllids increased from week 0 to week 2 of the incubation period before reaching a plateau with copy numbers comparable to those in psyllids from Lso-infected colonies. By week 3 of the incubation period, Lso was observed in the salivary glands of about 40% of the psyllids, which is consistent with fluorescence *in situ* hybridization observations made by Cooper et al. [15]. Results of the present study indicate that at least a two-week latent period is required for Lso multiplication and movement in potato psyllid adults and for the insects to successfully transmit the bacterium to new host plants. Findings from this study also indicate that Lso copy numbers greater than 10,000 and colonization of the salivary glands by Lso are required for psyllids to effectively transmit the bacterium. A closely related pathogen, “*Ca.* Liberibacter asiaticus,” transmitted by the Asian citrus psyllid also multiplies in the vector and colonizes its salivary glands, but the latent period required for the citrus psyllid to become infective is not known [23], [26], [27].

Upon analyzing the Lso haplotype in the psyllids used in this study, haplotypes A and B were detected in 81 and 17% of the insects, respectively. It is important to note however that no significant difference was observed in latent period of Lso in the psyllids between the two haplotypes. Additionally, while four different psyllid haplotypes exist [10], [13], all psyllids used in this study were of the Central haplotype. Psyllids of this Central haplotype have been identified in the Central United States, where the zebra chip disease was initially reported in the United States in the early 2000s, and where growers have experienced the economically devastating effects of the disease [4].

Although 60–80% of psyllids tested positive for Lso using qPCR, Lso was observed in less than 50% of psyllid salivary glands by week 3 of the incubation period. Less than 40% of plants that were exposed to psyllids for 24 h became infected with Lso. These findings are consistent with a previous study that used ‘Ranger Russet’ potato as a host to test infectivity of psyllids [15]. That study indicated that 100% of psyllids tested positive for Lso by PCR, but only 40% of psyllids had infected salivary glands, and less than 60% of plants inoculated with psyllids became infected with Lso. Several factors could potentially account for these inconsistencies in Lso infection rates in psyllids and Lso transmission rates by psyllids. First, our methods detect the establishment of Lso in plants, not necessarily the transmission rate by psyllids. Therefore, the actual transmission rates might be higher if plant defenses [28]–[30] occasionally prevent Lso infection or prevent Lso from moving from the initial inoculation site. Second, insect immune systems or other barriers within the insects may prevent the pathogen from either passing through the midgut wall or colonizing the salivary glands [15]. Finally, the methods of detecting Lso in insects or plants used in this study may fail to detect low levels of Lso infection. Significantly fewer psyllids tested positive for Lso on week 0 of the incubation period compared with weeks 1, 2 and 3 indicating that qPCR failed to detect Lso in some of the psyllids on week 0. It is possible that Lso is present in more host plants than observed, but below detectable levels, and the relationship between Lso infection and development of symptoms in plants is not well understood. Detection of Lso using fluorescent *in situ* hybridization is not perfect either because tissues or partial tissue samples are sometimes lost. Therefore, infection of salivary glands was likely higher than observed.

In summary, under the conditions used in this study, we indicate that a two-week latent period at temperatures ranging from 24–28°C is required for Lso to multiply and colonize the salivary glands of potato psyllids and for these insects to effectively transmit the bacterium to new host plants. These findings based on qPCR, fluorescence *in situ* hybridization, and the potato as a biological assay host suggest that acquisition and transmission of Lso by potato psyllids follows a pattern consistent with a propagative, circulative, and persistent mode of transmission [31]–[33]. The documentation of patterns in Lso acquisition and transmission by the potato psyllid contributes to a better understanding of the epidemiology of this bacterium and related diseases. This knowledge should improve the interpretation of results from field studies investigating Lso transmission among host plants. Furthermore, this information could improve the efficiency of monitoring infective psyllid populations and of timing insecticide applications to prevent zebra chip and other potato psyllid-transmitted diseases. Since only psyllids of the Central haplotype were used in the present study, additional research is needed to investigate interactions between the different haplotypes of Lso and those of the potato psyllid.
